# Simultaneous ultra-high frequency photoacoustic microscopy and photoacoustic radiometry of zebrafish larvae *in vivo*

**DOI:** 10.1016/j.pacs.2018.08.004

**Published:** 2018-09-01

**Authors:** Michael J. Moore, Suzan El-Rass, Yongliang Xiao, Youdong Wang, Xiao-Yan Wen, Michael C. Kolios

**Affiliations:** a Department of Physics, Ryerson University, Toronto, M5B 2K3, Canada; b Institute for Biomedical Engineering and Science Technology, A Partnership Between Ryerson University and St. Michael’s Hospital, Toronto, M5B 1W8, Canada; c Keenan Research Center for Biomedical Science, Li Ka Shing Knowledge Institute, St Michael’s Hospital, Toronto, M5B 1W8, Canada; d Zebrafish Centre for Advanced Drug Discovery, Li Ka Shing Knowledge Institute, St Michael’s Hospital, Toronto, M5B 1W8, Canada; e Institute of Medical Science, Departments of Medicine, Laboratory Medicine and Pathobiology & Physiology, University of Toronto, Toronto, M5S 1A1, Canada

**Keywords:** Label-free, Vasculature, Angiogenesis, Photoacoustic radiometry

## Abstract

With their optically transparent appearance, zebrafish larvae are readily imaged with optical-resolution photoacoustic (PA) microscopy (OR-PAM). Previous OR-PAM studies have mapped endogenous chromophores (e.g. melanin and hemoglobin) within larvae; however, anatomical features cannot be imaged with OR-PAM alone due to insufficient optical absorption. We have previously reported on the photoacoustic radiometry (PAR) technique, which can be used simultaneously with OR-PAM to generate images dependent upon the optical attenuation properties of a sample. Here we demonstrate application of the duplex PAR/PA technique for label-free imaging of the anatomy and vasculature of zebrafish larvae in vivo at 200 and 400 MHz ultrasound detection frequencies. We then use the technique to assess the effects of anti-angiogenic drugs on the development of the larval vasculature. Our results demonstrate the effectiveness of simultaneous PAR/PA for acquiring anatomical images of optically transparent samples in vivo, and its potential applications in assessing drug efficacy and embryonic development.

## Graphical abstract

**Figure:**
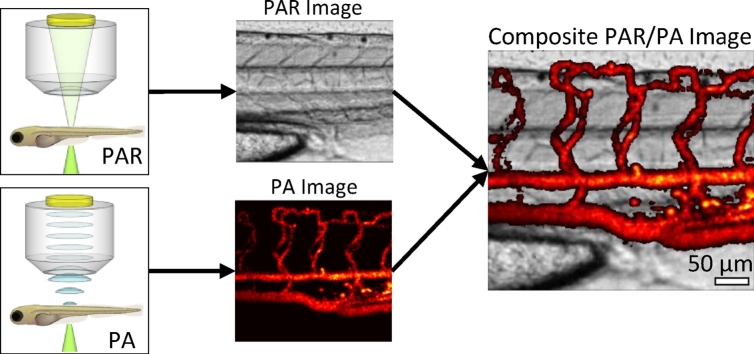

## 1 Introduction

In recent years, there has been a rapid increase in the use of zebrafish as specimens for biomedical research [1]. The rapid adoption of zebrafish as an in vivo biological model has been facilitated by several factors, including their low cost, high fecundity, and short maturation time [2,3]. As small animal models, zebrafish have been used in studies investigating the progression of disease [4], drug screening and discovery [1,5], and embryonic development [[5], [6], [7]]. While adult wild-type zebrafish exhibit a characteristic striped patterning dictated by the three variants of pigmentation cells in their bodies (melanophores, xanthophores, and iridophores) [8,9], the larval fish are largely transparent, and their internal structures are easily viewed using conventional microscopy techniques. To further enhance optical clarity, chemicals such as 1phenyl 2-thiourea (PTU) can be added early in the developmental cycle to inhibit melanophore production [10], or mutant fish which express limited pigmentation into adulthood are used [11].

Coupled with high optical transparency and abundant endogenous chromophores (i.e. melanin and hemoglobin) zebrafish larvae are also excellent candidates for imaging with photoacoustic microscopy (PAM). Conventional transmission-mode optical-resolution photoacoustic microscopy (OR-PAM) approaches have been used to image the eye, vascular system, and heart [12] as well as cardio-cerebrovascular development [13] in vivo in pigment-supressed zebrafish larvae. Other techniques, such as spatial resolution-invariant PAM (SIRPAM) have been used to acquire whole-body images of pigmentation in 3 day-post-fertilization (dpf) larvae over a lateral resolution-invariant axial range of 1.8 mm [14]. Multiview imaging techniques have also been employed in both PAM [15] and photoacoustic mesoscopy [16] setups to generate images with more isotropic resolution; however, these studies have only been performed with *ex vivo* specimens.

While PAM techniques yield high resolution maps of endogenous absorption, they contain no information pertaining to the gross anatomical tissue of the zebrafish, which has negligible absorption at illumination wavelengths typically used in PAM. For this reason, recent work has focused on creating hybrid systems combining several types of imaging modalities (e.g. as is commonly done with fluorescence and brightfield microscopy) with PAM to provide additional context for the acquired images. Rao et al. used an integrated photoacoustic, confocal, and two-photon microscope to acquire confocal fluorescence images of the spinal cord and posterior lateral line nerve as well as PAM images of vasculature in vivo in a transgenic larvae [17]. Soliman et al. used an integrated multi-photon and multi-scale photoacoustic microscope to acquire label free OR-PAM, as well as brightfield, and second, and third harmonic generation microscopy images of a 6 dpf wild-type zebrafish larvae [18]. The hybrid system was able to resolve melanin spots with PAM, as well as individual muscle fibrils and connective tissue within the trunk of the embryo with second and third harmonic generation microscopy, respectively. Finally, PAM systems incorporating optical coherence tomography detection have been used to image features including the larval eye, yolk, and swim bladder [19]. While each of these systems is capable of producing striking composite images depicting several unique anatomical features, each system requires specialized optical setups and the use of more than one laser. Furthermore, due to the different illumination pathways employed, no system was capable of simultaneous image acquisition.

We recently reported on a technique, termed photoacoustic radiometry (PAR), that can be used to simultaneously generate PA images as well as optical attenuation images using conventional transmission style OR-PAM [20,21]. In brief, in PAR imaging each laser pulse that is used to generate a PA signal is attenuated as it propagates through the sample. The attenuated optical pulse then impinges upon metallic components of the ultrasound (US) transducer - generating an additional PA signal within it that can be gated and used to reconstruct images. The amplitude of the PAR signal is directly proportional to the amount of light which is transmitted through the sample and incident on the transducer [20,21]. Attenuation of the laser beam due to both optical absorption and scattering decreases the signal amplitude, creating unique contrast in the resultant PAR images. Furthermore, since both PAR and PA signals are generated from a single laser pulse, both signals are acquired in the same RF-line and yield simultaneous co-registered images.

In this work, we demonstrate simultaneous label-free PAR and PA imaging of live zebrafish larvae. PAR images reveal structural features of the gross anatomy, while volumetric PA images depicted single red blood cells (RBC) and the vascular morphology. When merged, the PAR images provide useful landmarks for identifying the vasculature in the PA image. Finally, we applied the technique to study the effects of an anti-angiogenic drug that hinders the development of larval vasculature.

## 2 Methods

### 2.1 System setup

**Fig. 1:**
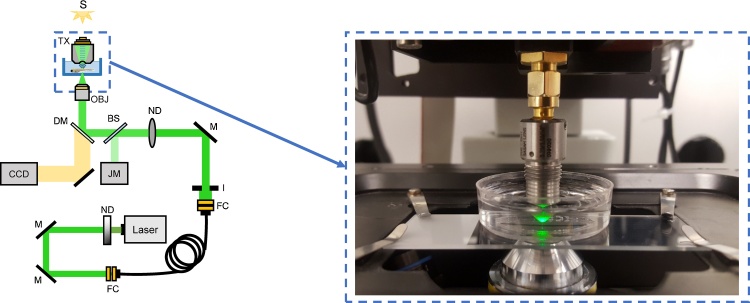
Schematic of the experimental setup. A photograph of the components in the dashed box on the left is shown on the right hand side of the image. For fluorescence imaging, the turret housing the dichroic mirror (DM) is rotated, placing the fluorescence cube (not shown) in the optical path instead of the DM. Abbreviations: ND, neutral density filter; M, mirror; FC, fiber coupler; I, iris; BS, beamsplitter; JM, joulemeter; OBJ, objective; TX, transducer; S, brightfield light source.

A schematic of the experimental system is shown in Fig. 1. A SASAM (SAarland Scanning Acoustic Microscope) photoacoustic microscope (Kibero, Saarbruken) built on an Olympus IX-81 microscope modified to include a pulsed 532 nm laser with pulse repetition frequency (PRF) of 4 kHz and pulse width of 330 ps (TeemPhotonics, France) was used for sample scanning. The laser was directed through a variable optical density neutral density (ND) filter and coupled into a single-mode fiber with a 2.5 μm core diameter and NA of 0.13 (Coastal Connections, USA). The collimated laser beam at the fiber output was spatially filtered by an iris and passed through a second ND filter. A portion of the beam was sampled using a 70/30 (T/R) beamsplitter (Thorlabs, USA) and directed to a joulemeter for pulse-to-pulse energy measurement (Gentec-EO, Canada). The transmitted portion of the beam was directed through the right side port of the SASAM and reflected off a dichroic mirror (Chroma Technology Corp., USA) housed in the IX-81 fluorescence cube turret. It was then focused through a 4X optical objective with a NA of 0.1 (Olympus, Japan) onto the sample. The laser pulse energy after the objective was approximately 50 nJ. The profile of the laser beam was measured using PAR with a knife-edge technique (Supplementary Fig. 1), and the full-width at half-maximum (FWHM) of the beam at the focal spot was found to be 5.3 μm. Fluorescence images of the larvae were acquired by rotating the turret to a fluorescence cube with excitation and emission wavelengths of 480 nm and 520 nm, respectively, and using a CCD camera affixed to the IX-81 left side port for image acquisition. Two different single element transducers were used in this work: a 200 MHz transducer with -6 dB bandwidth of 120 MHz, and a 400 MHz transducer with a -6 dB bandwidth of 180 MHz. The lateral/axial resolution of the transducers were calculated to be 8 μm/11 μm for the 200 MHz transducer, and 4 μm/7.5 μm for the 400 MHz transducer. The zebrafish larvae were placed on a motorized sample stage (Marzhauser Wetzlar, Germany), and scanned through the overlapping laser/transducer focal spots in a raster pattern. The step size in both the fast and slow scanning directions was 2 μm. PAR signals generated within the transducer and PA signals arriving from the sample were acquired in the same RF-line. Acquired RF-lines were digitized at a rate of 8 Gs/s using a 10 bit digitizer (Acquiris, USA), and were averaged 150 times to increase the SNR.

### 2.2 Preparation of zebrafish larvae

The zebrafish were raised using the techniques outlined in [22]. For the present study, two variants of zebrafish larvae were used: the transgenic *Tg* (*flk1*:GFP) line, which exhibits green fluorescent protein (GFP) expressing endothelial cells [23]; and the mutant *casper* line, which is largely devoid of melanophores and iridophores [11]. All zebrafish strains were housed and maintained under standard husbandry conditions [3]. To prevent excess melanin production in the *Tg* (*flk1*:GFP) fish, the embryos were treated with 200 μM of PTU [10] at 10 h post fertilization (hpf). For fish treated with the anti-angiogenic drug indirubin-3′-monoxime (I3M, or IRO), 4 μM was added to the zebrafish egg water at 8 hpf. All zebrafish experiments were conducted in accordance with St. Michael’s Hospital Animal Care Committee approved protocol ACC403.

A glass-bottom petri dish (MatTek, USA) was filled with 300 μL of molten 1.5% low melting point agarose (Sigma, USA) at 40 °C. The agarose was allowed to set in a 4 °C fridge for 15 min. The zebrafish larvae were anesthetized using a 0.003% (w/v) solution of tricane (Sigma, USA), and pipetted onto the agarose filled petri dishes. Excess egg water was aspirated, and the fish was covered with 20 μL of the molten 40 °C agarose. The dishes containing larvae were left to set at room temperature for 30 min prior to imaging.

### 2.3 Image formation

The acquired 3D scan datasets were time gated to isolate the PAR and PA signals. The signal envelope for each RF-line was computed, and maximum amplitude projection (MAP) images were generated separately for both the PAR and PA data. For display purposes, the MAP images were smoothed using a 2D Gaussian smoothing filter and interpolated. Prior to smoothing, the PA MAP images from scans of *Tg* (*flk1*:GFP) fish were log compressed, thresholded, and a dilate mask (disk structuring element, radius 1 pixel) was applied to enhance visualization of the vasculature.

## 3 Results & discussion

### 3.1 In vivo imaging of transgenic zebrafish

**Fig. 2:**
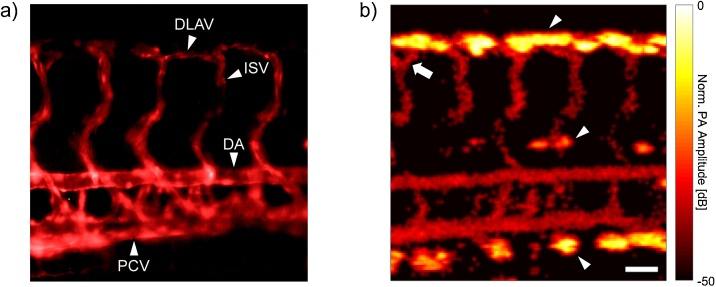
**a)** Fluorescence image of the GFP expressing endothelial cells in the trunk of a *Tg* (*flk1*:GFP) larval zebrafish. **b)** PA image of the same fish. Erythrocytes within the lumenized vessels allow for visualization of perfused vasculature. The arrow denotes a bifurcation in one of the ISVs, while the arrowheads indicate melanin spots. The scale bar is 50 μm and can be applied to both images. DLAV = Dorsal longitudinal anastomotic vessel, ISV = Intersegmental vessel, DA = Dorsal aorta, PCV = Posterior cardinal vein.

To our best knowledge, there has been no direct comparison of PA images of the developing zebrafish vasculature with fluorescence microscopy images of the vasculature from the same larva. Towards this end, we acquired fluorescence and PAM images of the same 4 dpf *Tg* (*flk1*:GFP) zebrafish larvae. Fig. 2a shows the GFP expressing endothelial cells in the larval zebrafish trunk. The vessels have been pseudo-coloured red to aid with comparison to the PA image. At this stage of development, the most prominent vessels in the trunk are the dorsal aorta (DA) and the posterior cardinal vein (PCV), which run horizontally, and the vertical intersegmental vessels (ISV), which merge at the dorsal side of the zebrafish to form the dorsal longitudinal anastomotic vessel (DLAV) [24]. Fig. 2b shows the corresponding PA MAP image of the same scan region acquired with the 400 MHz transducer. There is good agreement between the vasculature presented in the fluorescence and PA MAP image, and fine structure like the presence of a bifurcation in one of the ISV’s (indicated with an arrow) is preserved. Although the fish was exposed to PTU early in its development, the presence of strongly absorbing melanophores is noted in the PA image (arrowheads). The signal from the melanin was upwards of 30 dB greater than the signal from the vessels, which may explain why the vasculature is not readily discernable in other PAM scans of wild type embryos [14,15]. When comparing the two images, it appears that PA is unable to resolve several of the vessels present in the fluorescence image, most noticeably the ones between the DA and PCV. However, while both images provide indication of the morphology of the vasculature, the origin of the contrast in either is fundamentally different. The signal in the fluorescence image is due to GFP sequestered within the endothelial cells, and as such does not discriminate between partially developed and functional vasculature. In contrast, the signal in the PA image originates from RBCs within the vasculature, and therefore, shows only fully formed vessels with lumens that are capable of supporting blood flow (as assessed by RBC transit through the vessel). Taken together, these images provide a more complete overview of the development of the embryonic cardiovascular system, which would otherwise only be possible with double transgenic zebrafish lines expressing fluorescent proteins in both their endothelial cells and RBCs [25].

In contrast to the homogeneous appearance of the melanophores, the vessels in the PA image have a granular texture, which is most prominent in the ISVs and to a lesser extent in the DA and PCV. The graininess is a result of the motion of the circulating RBCs. When scanning over a vessel, the total number of excited PA sources (i.e. RBCs) within the confocal detection region fluctuates as a function of time, modulating the amplitude of the resultant PA signal. In regions where the RBC flow is sporadic and single file (e.g. the ISVs), it is not guaranteed that any RBCs will enter the excitation zone during the transducer dwell time, resulting in no detectable signal at that segment of the vessel. The effects of discontinuous blood flow on the appearance of PAM images of mouse ear vasculature have also been noted [26], but are not as prominent due to the much larger scan regions considered. While it would seem that the granular appearance of the PA image is a disadvantage of the technique, it may in fact present an opportunity to further enhance image resolution. Recently, Chaigne et al. reported a new technique which uses statistical analysis of fluctuations in PA signals resulting from absorber motion for super-resolution optical fluctuation imaging (SOFI) [27]. In contrast to superresolution US techniques [28], the PA SOFI approach does not require extremely sparse source distribution, and is thus ideal when imaging samples with endogenous sources like RBCs in the microvasculature. Application of the technique to the datasets here (either with endogenous or exogenous contrast agents) may help to further improve resolution of small vessels and reduce background noise.

### 3.2 Simultaneous duplex imaging of zebrafish larvae

**Fig. 3:**
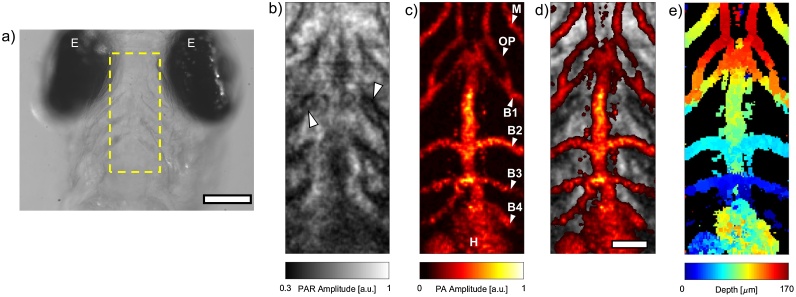
**a)** Brightfield image of the head of a *casper* zebrafish larva. The dashed box indicates the scan ROI. **b)** PAR image of the ROI. Arrowheads denote the pharyngeal arches. **c)** PA image acquired from the same dataset used to generate b). The presence of several vessels & the heart is noted. **d)** Composite PAR & PA image. **e)** Depth map of PA image. The scale bars in a) and d) are 150 μm and 50 μm, respectively. E = Eye, M = Mandibular arch, OP = Opercular artery, B1-4 = Branchial arches 1-4, H = Heart.

To demonstrate our duplex PAR/PA imaging technique, we then imaged 5 dpf mutant *casper* zebrafish larvae with the 200 MHz transducer. A brightfield image of the coronal view of a larval zebrafish head is shown in Fig. 3a. To optimize contrast in the resultant PAR/PA images, we targeted the central region of the head between the eyes (indicated with a dashed box). The simultaneously acquired PAR and PA images are shown in Fig. 3b and c, respectively. The contrast in the PAR image is due to both absorption and scattering of the excitation laser beam as it passes through the head, with dark regions indicating increased optical attenuation. There is good agreement of anatomical features between the brightfield and PAR images. The most apparent are the pharyngeal arches, which are observed at the periphery of the scan region (denoted with arrowheads). These cartilaginous structures offer support for the developing zebrafish jaw, and for the gills which will form between them [29]. The PA scan shown in Fig. 3c was generated from the same scan data as in Fig. 3b. As in Fig. 2b, the vessels have a granular appearance indicative of high blood flow. The scan reveals a symmetric distribution of vessels to either side of the image, with the heart at the bottom. The mandibular arch (M), opercular artery (OP), and branchial arches 1–4 (B1-4) are readily identifiable upon comparison to literature [24]. As both the PAR and PA images are acquired simultaneously, the two images can easily be overlaid without the need for image scaling or co-registration. An overlay of the PAR and PA images is shown in Fig. 3d. The overlay provides useful landmarks and context for identifying features in the PA image; for example, demonstrating the expected interleaving of the aortic arches between adjacent pairs of pharyngeal arches [29].

One of the advantages of using ultra high frequency (UHF) transducers is the fine lateral and axial resolution due to the high central frequency and large bandwidth, respectively [[30], [31], [32]]. This enables generation of high-resolution volumetric representations of the vascular data (Supplementary Movie 1) as well as striking depth-encoded images, such as the one shown in Fig. 3e. The depth-encoded image displays the relative depth at which the maximal PA signal is found; structures which are closer to the transducer appear as dark blue, while those which are deeper within the larvae appear red. The results here indicate that the arches which are more distal from the heart slope down toward the ventral side of the fish, with the exception of the mandibular arches, which loop back towards the heart and the dorsal side. These results again can be verified by comparison with the expected vascular structure (cf. Fig. 6E in [24]).

**Fig. 4:**
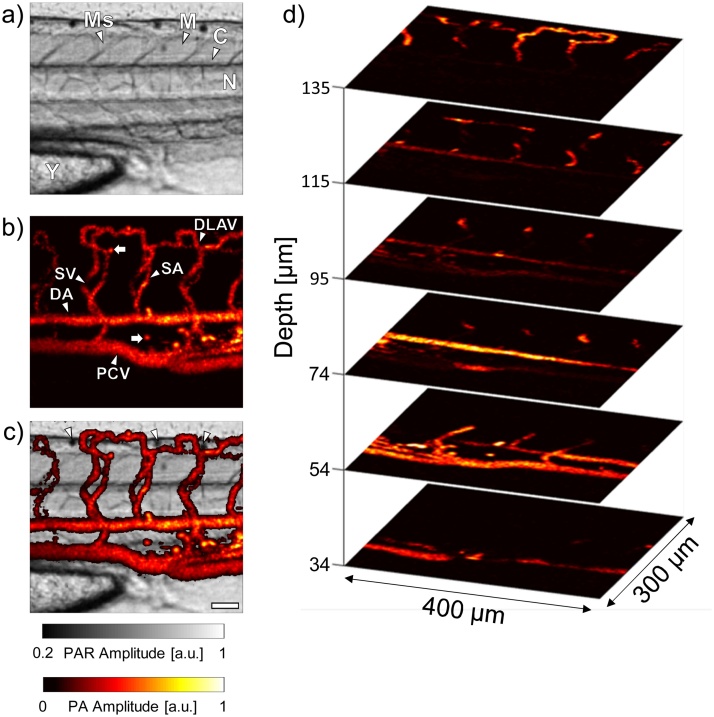
**a)** PAR image of the trunk of a 5 dpf *casper* zebrafish. **b)** PA image of the trunk vasculature acquired simultaneously with a). **c)** Composite PAR/PA image. **d)** PA images at different depths within the larva. The scale bar in c) is 50 μm. Ms = Myosepta, M = Myotome, C = Central canal, N = Notochord, Y = Yolk, DLAV = Dorsal longitudinal anastomotic vessel, SV = Intersegmental vein, SA = Intersegmental artery, DA = Dorsal aorta, PCV = Posterior cardinal vein.

A scan of the trunk of a second live 5 dpf larvae, with the fish’s sagittal plane orthogonal to the 200 MHz transducer, was then performed. The resultant PAR image is shown in Fig. 4a. The image provides exceptional visualization of the different anatomical structures within the trunk. The rhomboid muscle segments of the trunk (myotomes, M) are separated by the connective tissue of the vertical myosepta (Ms) [29]. The notochord (N), which serves as the primary support structure for the larval fish, is clearly visible, with a slight indication of the central canal (C) segment of the neural tube running immediately dorsal. Finally, the yolk (Y) is readily identified at the ventral side of the fish due to its thick contouring and granular appearance.

The simultaneously acquired PA image is shown in Fig. 4b. To demonstrate the effects of postprocessing on the appearance of the PA MAP image, the raw MAP image, as well as the smoothed and smoothed then interpolated MAP images are shown in Supplementary Fig. 2. As in the case of the transgenic fish, the DA, PCV, ISVs – which have been separated into intersegmental arteries (SA) and veins (SV), and DLAV are all clearly identifiable. The SA and SV can be identified by their origin point, which is either on the DA or PCV, respectively. During angiogenesis, endothelial cells sprout from the DA near the myosepta to form the SA and DLAV; however, most have not yet lumenized and thus do not support circulation [33]. After these structures are established (at approximately 1.5 dpf), secondary sprouts emerge from the PCV and grow dorsally. In cases where these sprouts join with the SAs, the original connection between the SA and DA is severed, and the vessel, now identified as a SV, supports circulatory flow between the DA and the PCV [33]. The presence of strongly absorbing objects (denoted by arrows) is noted in the interstitial space, especially between the DA and PCV in the so called caudal hematopoietic tissue (CHT), which is an active region of hematopoiesis in the larval fish after 2 dpf [[34], [35], [36]]. Since the *casper* zebrafish are devoid of melanophores, we believe that these objects are individual RBCs. Zebrafish RBCs are elliptical in shape and have minor and major axes of approximately 7 and 10 μm, respectively [37]. Lateral and axial profiles taken through the centre of one of the suspected RBCs are shown in Supplementary Fig. 3. The FWHMs of the profiles are 10 μm (lateral) and 12 μm (axial), and are consistent with the expected zebrafish RBC dimensions. These results demonstrate the potential of the UHF PA technique for resolving individual cells in vivo. Owing to the fine lateral and axial resolution, 3D visualizations of the trunk vasculature are also possible. A volumetric reconstruction of the PA data is shown in Supplementary Movie 2. Sagittal planes within the 3D PA dataset are presented in Fig. 4d, demonstrating the arrangement of the functional trunk vasculature. Each slice is separated by ∼20 μm in the axial direction. In addition to making it easier to identify vasculature at different planes in the fish, the slices further demonstrate that the fish is tilted with respect to the transducer, with the PCV being closest and the DLAV furthest away.

As seen in Supplementary Movie 2, the ISVs form a shape reminiscent of a ‘Figure-8’ in the transverse cross section. The overlay of the PAR and PA images shown in Fig. 4c provides context for this configuration. With the overlay, it can be seen that the ISVs must be superficially rerouted around both the notochord (which is immediately dorsal to the DA) and the neural tube, which is further dorsal to the notochord. Without the landmarks provided in the PAR image, histological techniques, which require fixation of the fish, would be required to obtain these insights. Of particular interest in the overlaid image are three dark spots (arrowheads). As evidenced by the PAR image, these regions are highly attenuating to the incident laser beam; however, they were found to produce no detectable PA signal. We hypothesize that these dark spots are iridophores, which are a type of pigment cell responsible for the silvery appearance of the adult fish. Iridophores contain a large number of reflective crystalline platelets which effectively form a stack of thin-films that scatter incident light [38,39]. It should be noted that *casper* zebrafish are expected to be completely devoid of iridophores [11]; however, several observations of the images in this and the proceeding section suggest that this may not always be the case. First, the location of the suspected iridophores in the PAR image is the dorsal side of the neural tube, which is where pigmentation cells develop in the larvae before migrating to their final location in the body [8,38]. Secondly, owing to their optical properties, iridophores were expected to be sources of strong contrast in PAR images but undetectable in PAM, consistent with Fig. 4a and b. Finally, the brightfield images of an equivalent dark region in the PAR image of a different *casper* zebrafish taken with the transducer blocking the central beam of the light source reveals that these spots are highly reflective. This is consistent with the expected appearance of the larval zebrafish iridophores under epi-illumination conditions (cf. Fig. 1, Fig. 3 in [40]).

### 3.3 Inhibition of vascular growth

**Fig. 5:**
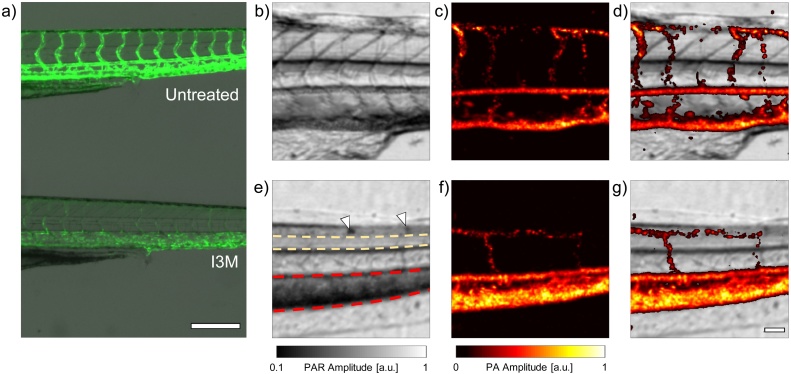
**a)** Fluorescence image of vasculature in the trunk of an untreated (top) and I3M treated (bottom) *Tg (flk1*:GFP*)* larval zebrafish. I3M is a potent inhibitor of angiogenesis, and fewer ISVs are formed in the treated fish. **b) – d)** PAR, PA, and composite images, respectively, of an untreated larva. Corresponding images for an I3M treated fish are shown in **e) – g)**. In e), arrowheads mark the location of iridophores, while the yellow contoured area is the neural tube, and the red contoured region has an excess of RBCs. The scale bar in a) is 250 μm. The scale bar in g) is 50 μm, and can be applied to b) – g).

It has previously been demonstrated that I3M is a potent inhibitor of ISV angiogenesis in larval zebrafish models [41,42]. As demonstrated in Fig. 5a, the number of ISVs in the trunk of the *Tg* (*flk1*:GFP) larvae decreases with increasing I3M concentration. Typically, transgenic zebrafish lines expressing fluorescent proteins in their endothelial cells must be used to observe the effects of the anti-angiogenic drug; however, it would be useful to assess these effects in the easily accessible wild type larvae also. Towards this end, we used the 200 MHz transducer to image I3M treated *casper* zebrafish larvae using the PAR/PA technique.

Simultaneously acquired PAR, PA, and composite images of an untreated larva are shown in Fig. 5b–d, respectively. Equivalent images of an I3M treated larva are shown in Figs. 5e–g. Comparing the PAR images, the myosepta are much more prominent in the untreated larva, while the neural tube (yellow contour) is more readily visible in the treated fish. As I3M only affects the developing vasculature, these differences could potentially be attributed to different focusing of the optical objective within the fish trunk. In the treated fish, a conspicuous dark region ventral to the notochord is observed (red contour), along with iridophores (arrowheads), whose presence is verified with brightfield microscopy (Supplementary Fig. 4). There appear to be two major differences in the appearance of the PA images of treated vs. untreated fish. First, as expected, the treated fish has fewer ISVs apparent in the scan ROI than the untreated fish. This is due to the fact that only ISVs which are capable of supporting circulatory blood flow are shown in the PA images. Vessels which are not fully developed, or which have not lumenized, due to the addition of I3M, are not visible. Second, while the appearance of the DA is similar in both images, the appearance of the PCV is markedly different even though both scans were acquired near the same anatomical location. One possible explanation for this is the expansion of the vessel to accommodate a larger RBC load due to loss of supporting ISV vasculature. In addition to showing the organization of the vasculature, the composite images reveal that the dark region noted in Fig. 5e is a result of the increased attenuation due to the enlarged PCV size.

It should be noted that I3M has a deep red colour, which dyes the zebrafish yolk sac [41] and causes it to produce strong PA signals (data not shown). This limits the PAR/PA technique to ROIs that are devoid of yolk, which would otherwise overwhelm the PA signal from the vessels. Nevertheless, the results of this preliminary investigation indicate that our dual modality technique could be an effective tool for assessing the effects of antiangiogenic drugs on zebrafish larvae. In the future we plan to assess the impact of higher I3M concentrations on both the anatomy and vasculature of developing wild type zebrafish, as well as investigate the effects of other drugs such as statins, which may be involved in vascular stability and intracerebral hemorrhages [[43], [44], [45]].

## 4 Conclusion

The PAR images made it possible to identify anatomical structures including the myotomes, notochord, and yolk; while the PA images made it possible to visualize the head and trunk vasculature with single cell resolution. While this represents the first time that simultaneous images of this nature have been acquired using only OR-PAM, there are some inherent limitations to our technique. First, as with all UHF studies, acoustic attenuation limits the depth at which high SNR signals can be acquired [30]. This restricts the described dual modality technique to whole-body imaging of larval fish, or to regions less than 100 μm deep at higher frequencies (e.g. 1 GHz) in mature fish. Another limitation of the current system is the scanning time. A 160 × 160 point raster scan currently takes approximately 30 min; however, we plan to reduce this scan time by incorporating a laser with a higher PRF. In summary, we have demonstrated a simultaneous label-free technique for imaging the anatomy and vasculature of transgenic and mutant *casper* zebrafish larvae in vivo with single cell resolution. We believe that our technique will find applications in studying gold nanoparticle aggregation in tissue, and the progression of cancer metastasis.

## Conflict of interest

MCK and MJM have financial interests in Echofos Medical Inc., which, however, did not support this work. The remaining authors declare no competing financial interests.
