# Supplementary material for: Simultaneous ultra-high frequency photoacoustic microscopy and photoacoustic radiometry of zebrafish larvae in vivo
Source: Photoacoustics. 2018 Sep 1;12:14–21. doi: 10.1016/j.pacs.2018.08.004 (PMC6139000; doi:10.1016/j.pacs.2018.08.004)
Supplement: Supplementary file 1 [file mmc1.pdf]

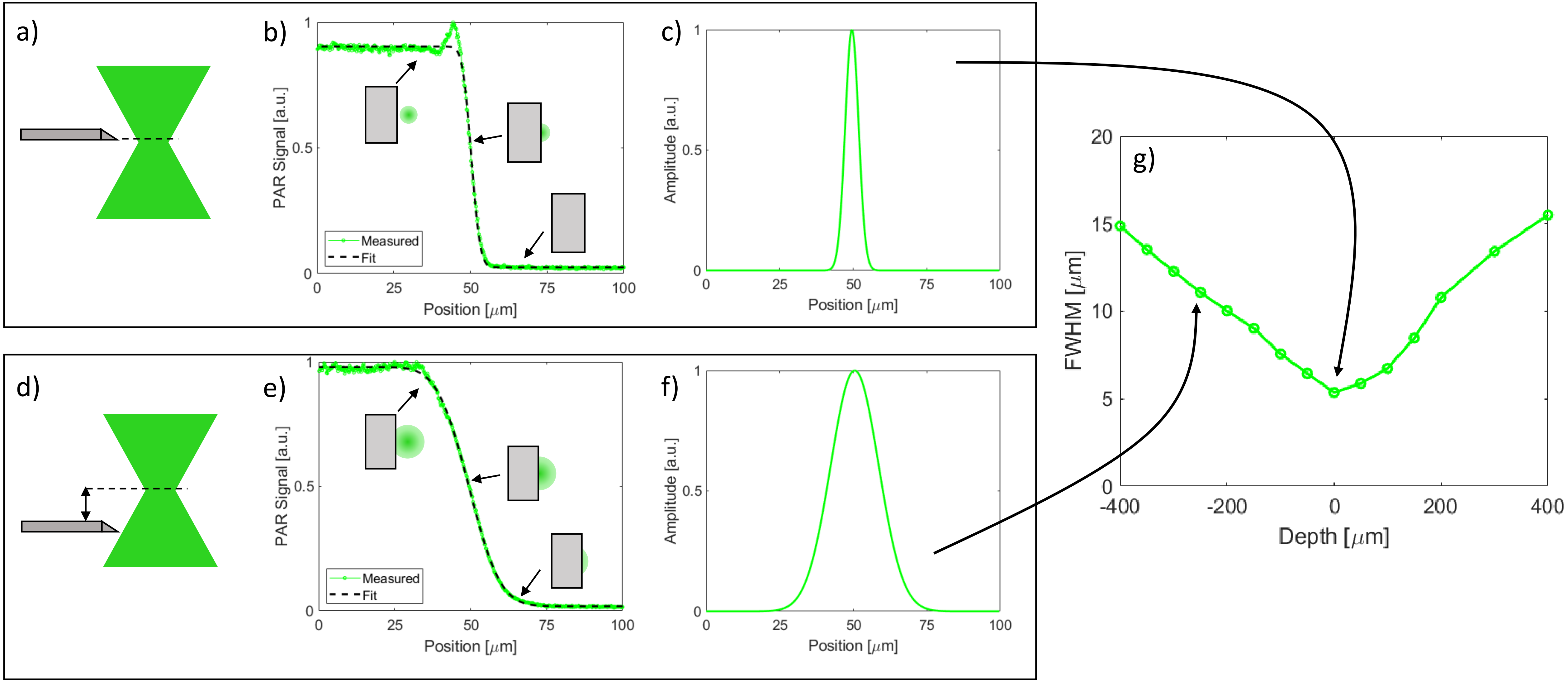

Supplementary Figure 1: Overview of the knife-edge technique for assessing the beam profile. A razor blade is translated through the focused laser beam at a specified axial depth. A schematic of the razorblade positioned at the beam waist **(a)** and 250 µm below the waist **(d)** are shown. Axial positioning of the laser beam is achieved by translating the focusing objective. **b) & e)** The PAR signal as a function of razor blade position is measured and the resulting datapoints fit to an erf function. The view of the razor blade (gray rectangle) along the propagation direction of the laser beam is shown at various scan positions. **c) & f)** The fit curves are differentiated, and the FWHM of the resultant Gaussian is found. **g)** The FWHM is plotted as a function of axial position within the laser beam. The FWHM at the beam waist for this experiment was 5.3 µm.

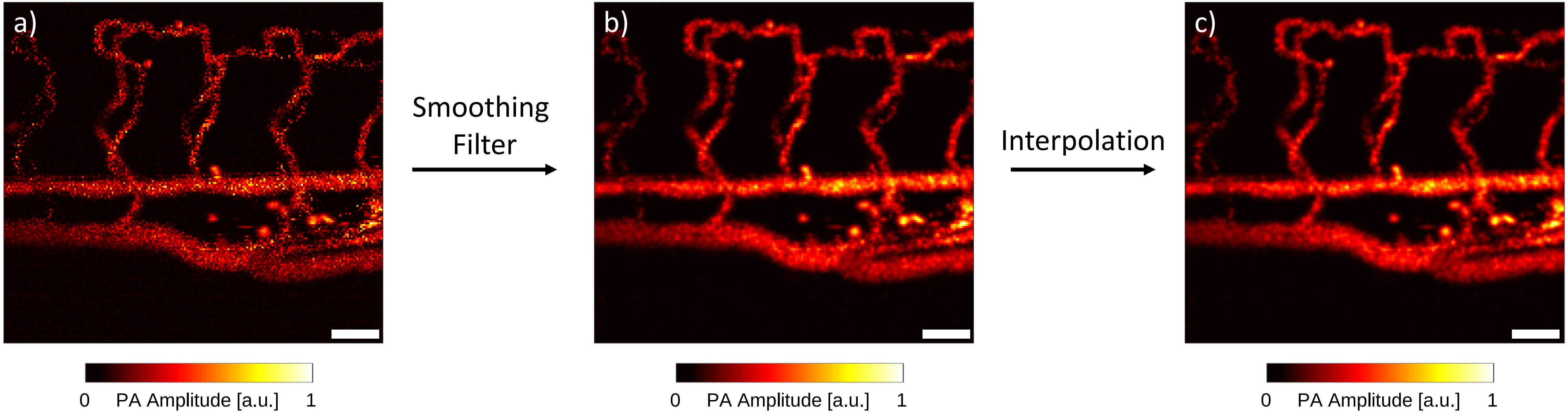

Supplementary Figure 2: a) The raw PA MAP image of the data used to generate Figure 4b. **b)** The image after application of a 2D Gaussian smoothing filter. The grainy appearance of the vessels in the image due to RBC motion is greatly reduced. **c)** The smoothed image after 1-point 2D interpolation. All scale bars are 50 µm.

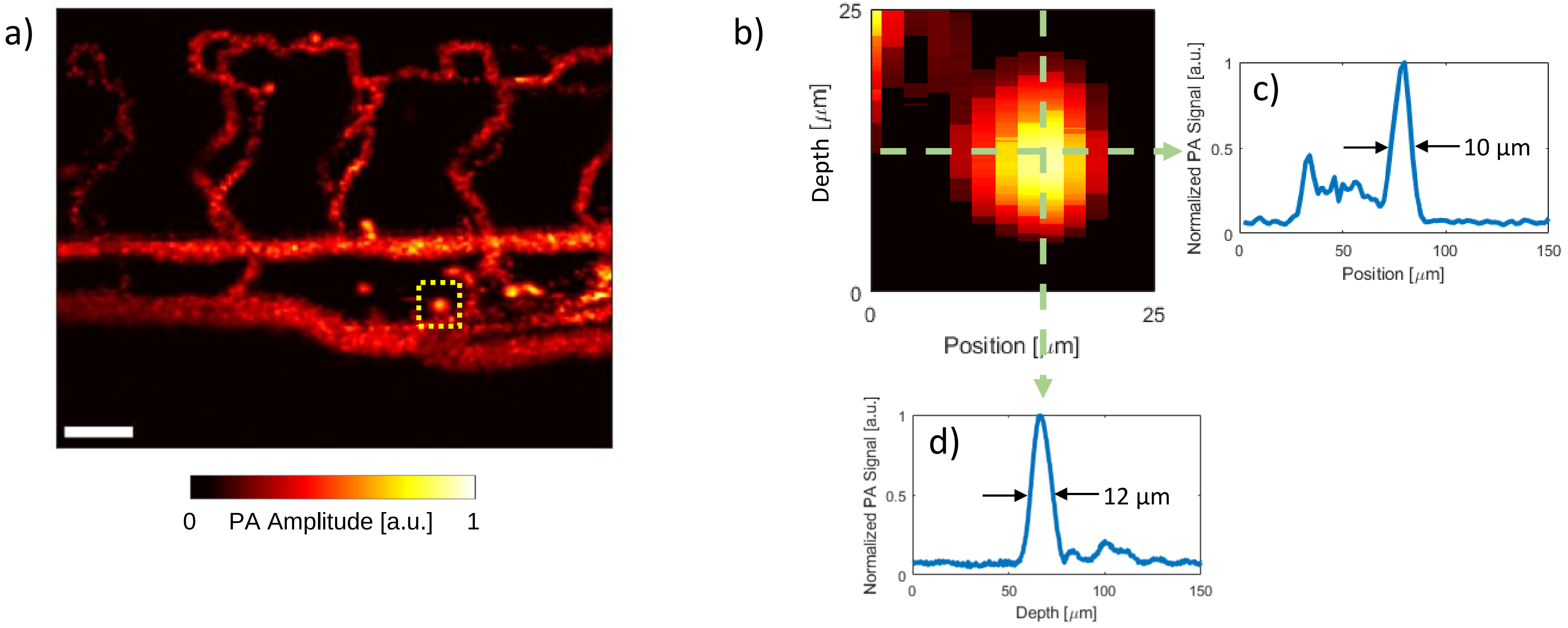

Supplementary Figure 3: a) PA MAP image from Figure 4b with a dashed region indicating a single RBC. **b)** The zoomed in B-Mode view of the same RBC in a). The lateral and axial profiles through the RBC are shown in **c)** and **d)**, respectively. The scale bar in a) is 50 µm.

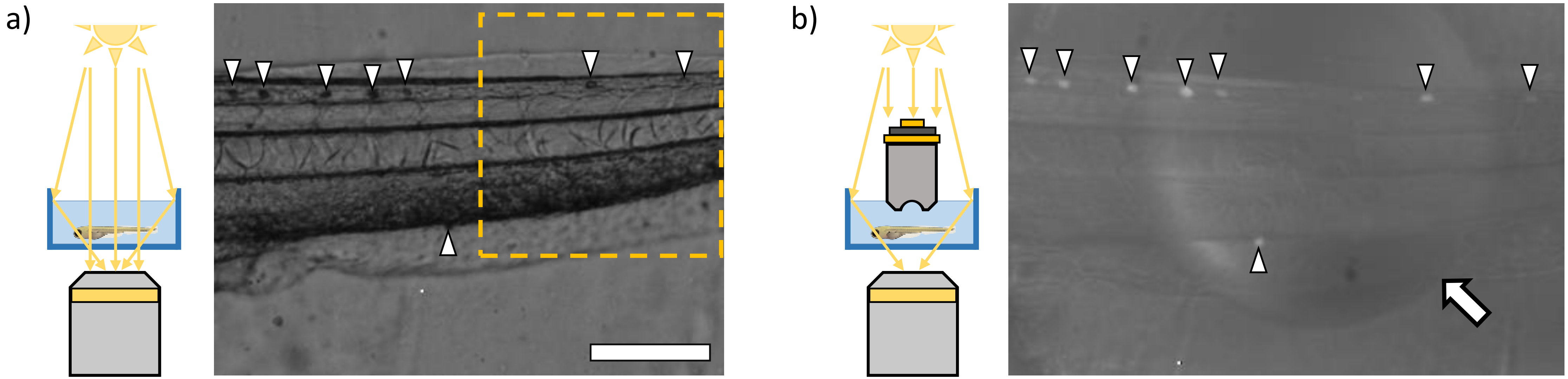

Supplementary Figure 4: Transmission brightfield microscopy images of a *casper* zebrafish without **(a)** and with **(b)** the US transducer in place between the sample & brightfield light source. With the transducer blocking the central portion of the light source, only oblique illumination occurs. This causes the scattering iridophores (arrowheads) to become highly visible. The dashed box in a) indicates the region scanned in Fig 5e-g). The rim of the transducer is indicated with an arrow in b). The scale bar in a) is 200 µm and can be applied to both images.

Supplementary Movie Captions

Supplementary Movie 1: 3D PA volume rendering of the vasculature depicted in Fig. 3c. The scale bar is 100 µm.

Supplementary Movie 2: 3D PA volume rendering of the zebrafish trunk vasculature depicted in Fig. 4b. The scale bar is 100 µm.
